# Reliability Estimation of Reinforced Slopes to Prioritize Maintenance Actions

**DOI:** 10.3390/ijerph18020373

**Published:** 2021-01-06

**Authors:** Farshad BahooToroody, Saeed Khalaj, Leonardo Leoni, Filippo De Carlo, Gianpaolo Di Bona, Antonio Forcina

**Affiliations:** 1 Department of Civil Engineering, University of Parsian, Qazvin 3176795591, Iran; farshadbh@gmail.com (F.B.); saeedkhalaj220@gmail.com (S.K.); 2 Department of Industrial Engineering (DIEF), University of Florence, 50123 Florence, Italy; leonardo.leoni1@stud.unifi.it; 3 Department of Civil and Mechanical Engineering, University of Cassino and Southern Lazio, 03043 Cassino, Italy; dibona@unicas.it; 4 Department of Engineering, University of Naples “Parthenope”, 80133 Naples, Italy; antonio.forcina@uniparthenope.it

**Keywords:** geotextile-reinforced slopes, failure modeling, drainage system, hierarchical Bayesian modeling

## Abstract

Geosynthetics are extensively utilized to improve the stability of geotechnical structures and slopes in urban areas. Among all existing geosynthetics, geotextiles are widely used to reinforce unstable slopes due to their capabilities in facilitating reinforcement and drainage. To reduce settlement and increase the bearing capacity and slope stability, the classical use of geotextiles in embankments has been suggested. However, several catastrophic events have been reported, including failures in slopes in the absence of geotextiles. Many researchers have studied the stability of geotextile-reinforced slopes (GRSs) by employing different methods (analytical models, numerical simulation, etc.). The presence of source-to-source uncertainty in the gathered data increases the complexity of evaluating the failure risk in GRSs since the uncertainty varies among them. Consequently, developing a sound methodology is necessary to alleviate the risk complexity. Our study sought to develop an advanced risk-based maintenance (RBM) methodology for prioritizing maintenance operations by addressing fluctuations that accompany event data. For this purpose, a hierarchical Bayesian approach (HBA) was applied to estimate the failure probabilities of GRSs. Using Markov chain Monte Carlo simulations of likelihood function and prior distribution, the HBA can incorporate the aforementioned uncertainties. The proposed method can be exploited by urban designers, asset managers, and policymakers to predict the mean time to failures, thus directly avoiding unnecessary maintenance and safety consequences. To demonstrate the application of the proposed methodology, the performance of nine reinforced slopes was considered. The results indicate that the average failure probability of the system in an hour is $2.8\times 10^{-5}$ during its lifespan, which shows that the proposed evaluation method is more realistic than the traditional methods.

## 1. Introduction

Soil slope stability analysis is a typical engineering issue that is widely associated with enormous geotechnical problems in practice. Artificial reinforcement, including the use of geosynthetics, is used to strengthen slopes expected to experience a failure in the event of a disturbance [1]. Due to slope instability, fatalities, injuries, property damage, economic disruption, and environmental degradation have been reported [2]. The International Geosynthetics Society (IGS 1996) defines a geosynthetic product as a planar, polymeric (synthetic or natural) material used in contact with soil/rock and/or any other geotechnical material in civil-engineering applications [3]. During the past three decades, the advantages of using geosynthetic reinforcements have been verified by researchers [4,5,6,7,8]. Geosynthetics improve soil conditions by providing tensile resistance, reducing ground-improvement costs, and simplifying construction procedures. Different types of geosynthetics are useful in solving geotechnical problems, including geotextiles, geogrids, geonets, and geomembranes. Among all geosynthetics, geotextiles have been demonstrated as a useful tool to reinforce unstable slopes and provide separation, filtration, drainage, and containment of contaminants [9]. Consequently, a comprehensive analysis was needed to evaluate the effect of geotextiles on reinforcement, drainage, and separation in slopes. When considering different geotextile applications in reinforcing slopes, drainage plays an inevitable role in preventing the slopes from experiencing a collapse [10]. The installation of adequate drainage systems to reduce pore water pressure accumulation within slopes is a significant task that should be conducted to ensure the stability of geotextile-reinforced slopes (GRSs) [11]. Due to the low permeability of fine soils, their draining capacity is low. Rainfall infiltration and the groundwater level can seriously threaten the performance of GRSs. Therefore, more than 170 failures of extreme deformation or collapse have been reported. [12]. Studies in the literature have demonstrated that constructing and installing proper and adequate geotextiles can extend a slope’s lifespan by alleviating the pore water pressure [13,14,15]. In general, geotextile reinforcement is assumed as an equivalent force on the slope. Limit equilibrium and limit analysis methods have been employed to examine the stability of slopes reinforced with geotextiles [16,17]. In addition, other numerical methods, as well as the finite element approach, have been chosen to assist in evaluating the final stability of reinforced slopes against total forces [18,19,20]. Many studies have been devoted to investigating the geotextile-reinforced behavior in centrifuge model tests [21,22,23]. The reinforced slope responses were determined by establishing several 1 *g* model tests in a centrifuge. The centrifuge technique provides a more realistic and applicable result in evaluating the stability of GRSs. An equivalent stress level in a small-scale model was established in the centrifuge to model the real condition [24]. Accordingly, the shortcomings associated with numerical approaches could be partially eliminated. Field observation and model tests can potentially investigate the slope failure mechanism and evaluate the geotextile-reinforced behavior [25]. All presented approaches are deterministic methods, in which any significant variables should have a specific value neglecting any variation. However, significant variables are associated with intrinsic uncertainties that affect the outcomes [26]. Thus, to overcome this shortcoming, probabilistic risk-based methods should be developed to minimize the probability of failure when considering uncertain variables with continuous variations [27].

Unlike classical statistical methods, Bayesian techniques are mainly used in probabilistic risk assessment of uncertain events, in a wide range of information types. They are also applicable in modeling sparse data, or when the correlation between them is difficult to determine [28,29,30]. Advances in Bayesian statistics, such as the development of hierarchical Bayesian modeling (HBM), have been adopted, since the HBM technique is capable of coping with source-to-source variability in data samples. Any of the considered slopes take into account the stability analysis as a source. While a specific failure process—including the evaluation of several failure probabilities for GRSs—is being monitored, the instinct uncertainties in any source can be quite different compared to another source. Accordingly, a source-to-source variability analysis, which is a treatment of variability that can exist among sources of data in GRS samples, should be carried out to obtain more realistic and accurate results. HBM can model the parameters with nonstationary data and make a nonlinear correlation among data using open-source Markov chain Monte Carlo (MCMC) sampling software packages, including OpenBUGS [31].

There is still a need to appropriately address the uncertainties associated with the acquired data for each event to introduce an informative prior distribution and possible observations for the event’s parameter of interest [32]. Our study sought to develop a probabilistic methodology for the reliability analysis of reinforced slopes, focusing on failures through the drainage system, which is an application of geotextiles. Implementing HBM, which can model the correlations among parameters, can lead to more accurate predictions and more reliable results. To this end, MCMC was adopted as a tool to assist in estimating the posterior probabilities. The results provide fundamental information that can be used in further risk-mitigation strategies to improve and boost the safety of urban infrastructures. Prioritizing maintenance efforts is essential to produce a cost-effective risk strategy while optimizing resource consumption. To demonstrate the application of the proposed methodology, nine GRSs in Tehran were studied.

### Hierarchical Bayesian Modeling (HBM)

**Figure 1:**
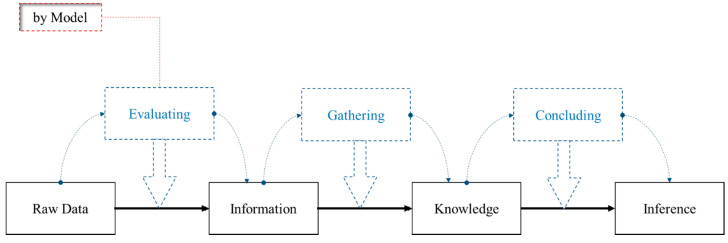
An overview of the inference process and its key elements [38].

The initial step in the performance of statistical inference is to acquire the observed values from a stochastic process defined as data that may incorporate various sources of uncertainty. The evaluation process that the developed model conducts using the raw data leads to obtaining information, from which knowledge can be gathered from the information. Finally, inference is based on a conclusion obtained from the knowledge [33]. HBM is a probabilistic approach that allows the organization of inference based on real-world conditions, and can assist in obtaining the posterior distribution of the parameters [34,35,36,37]. A brief overview of the inference performance procedure using data and the probabilistic model is shown in Figure 1.

In our study, the HBM was carried out based on Bayes’ theorem for carrying out Bayesian inference, which is given in Equation (1):

(1) $$\pi_{1}\left(\theta |x\right)=\;\frac{f\left(x|\theta \right)\pi_{0}\left(\theta \right)\;}{\int_{\theta }f\left(x|\theta \right)\pi_{0}\left(\theta \right)d\theta }$$

where θ is the unknown parameter of interest, f(x|θ) is the likelihood function, $\pi_{1}\left(\theta |x\right)$ is the posterior distribution of θ, and $\pi_{0}\left(\theta \right)$ is the prior distribution of θ. The hierarchical Bayesian framework is capable of calculating the multistage prior distribution for parameters of interest [39], and can be calculated as shown in Equation (2):

(2) $$\pi_{0}\left(\theta \right)=\;\int_{\emptyset }\pi_{1}\left(\theta |\varphi \right)\pi_{2}\left(\varphi \right)d\varphi$$

where $\pi_{1}\left(\theta |\varphi \right)$ is the first stage prior to the population variability in θ, $\pi_{2}\left(\varphi \right)$ is the hyperprior distribution representing the uncertainty in φ, while φ is a vector of hyperparameters. For instance, if φ=(α,β) follows a Weibull distribution, then α and β are the shape and scale parameter, respectively. To estimate the posterior distribution, the prior distribution, $\pi_{0}\left(\theta \right)$, should be modeled. This prior knowledge can be obtained from different sources, including numerical simulations [36], experiments [40], or industrial operations [41].

**Figure 2:**
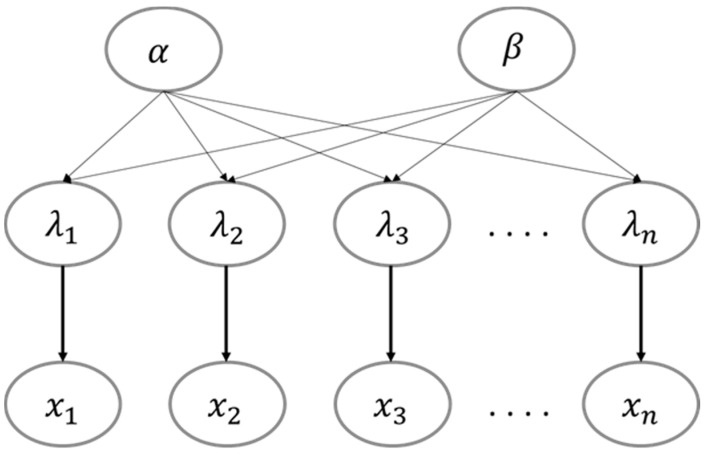
The directed acyclic graph of the hierarchical Bayesian modeling (HBM)**.**

The dependencies among different variables taken into account when establishing an HBM are shown in Figure 2. The posterior predictive distribution of parameter *λ*, which represents the source-to-source variability, is conditional upon a prior distribution introduced by α and β. Consequently, in each specific source, the marginal posterior distribution for *λ* is a continuous mixture of Gamma distributions, mixed over the posterior distribution of the hyperparameters α and β.

## 2. Developed Methodology

Every reinforced slope is expected to experience any type of failure during its lifespan. Thus, in the initial phase, different failure types and their relevant factors should be recognized. Among all identified factors, those with the same contribution in failure is selected as the main failure component. An integrated system reinforced with geotextiles is seen as a system with two main components: the slope and the reinforcing geotextiles. Our study modeled the failure probability of geotextile-reinforced slopes in the event of a failure of the drainage system. Choosing practical tools and validated assumptions results in more accurate and reliable results, which reduces the associated risks and uncertainties.

As recommended in [42], in the annual probability of failure in slope stability analysis, all the variables involved in the evaluation process should be considered constant over time. Therefore, in this study, a constant failure rate was considered to model the slope failure observations. According to the time-independent gathered data, the most efficient approach to estimate the failure rate of nonoverlapping interval events is to employ a homogeneous Poisson process (HPP). This model is useful when the inter-arrival time intervals between failures are independent and identically distributed according to the exponential distribution (*λ*). The time intervals between failure in an HPP are a sequence of independent and identical exponentially distributed random variables. The nonhomogeneous Poisson process (NHPP) can also be used to model the reliability of repairable systems. The difference between the HPP and the NHPP is that the rate of occurrence (intensity of events) varies with time in the NHPP, rather than being a constant as in the HPP. In the NHPP, the points are not exponentially distributed, but they are not independent samples from any other single distribution. Therefore, according to the previous studies [43,44,45] that used the HPP assumption, exponential distributions were adopted. Moreover, a Weibull distribution with a specific shape and scale parameter was established.

### Failure Modeling

Several probability models can be established to simulate the prior knowledge and likelihood function to model a failure given HPP assumption and using Bayesian inference. Here, the exponential distribution with the parameter of interest λ is employed to model the failures. To this end, as recommended in [38], a Gamma distribution can be adopted to present the prior distribution of λ. The likelihood function can be simulated by Poisson probability distribution, in which the number of failure events (x) is modeled in Equation (3):

(3) $$f\left(x|\lambda \right)=\frac{{\left(\lambda t\right)}^{x}e^{-\lambda t}}{x!},\;x=0,1,\ldots$$

where t is the exposure time and λ is the intensity of the Poisson distribution. To describe the variability of λ among all the assumed failure times, a gamma distribution should be adopted for hyperprior distributions, which are the probability models of hyperparameters. Thus, as shown in [28], in the following Equation, using the hyperparameters α and β, the first-stage prior distribution can be calculated using a gamma distribution.

(4) $$\pi_{1}\left(\lambda |\alpha ,\;\beta \right)=\frac{\beta^{\alpha }\lambda^{\alpha -1}e^{-\beta \lambda }}{\Gamma \left(\alpha \right)}$$

Before any further observation, the hyper-parameters are considered to be independent. Once the observations are recognized, the hyperparameters become dependent. According to the aforementioned dependency, the posterior distribution will be estimated [33].

As soon as the prior distributions and the likelihood functions are developed, the posterior distribution of the gamma parameters is anticipated by employing the MCMC simulations to assist. Additionally, the gamma prior was chosen because a gamma distribution is a conjugate prior for the Poisson distribution. Consequently, this predicted posterior distribution of λ can be set up in the exponential distribution to obtain the failure model.

## 3. Application to a Case Study

**Figure 3:**
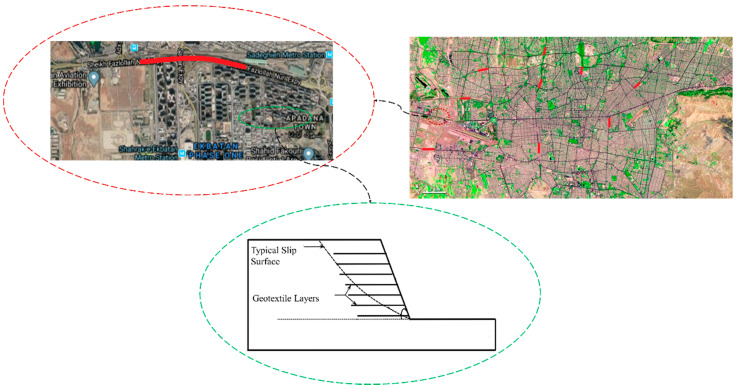
Graphical representation of the location of the case study.

To demonstrate the application of the developed methodology, a case study of nine GRSs in Tehran was considered (Figure 3). A poor drainage system may lead to a catastrophic failure in slopes during their lifetime. Therefore, these systems were considered as the cause of failure propagation that was examined using the proposed methodology.

### 3.1. Geometry of the GRSs

**Figure 4:**
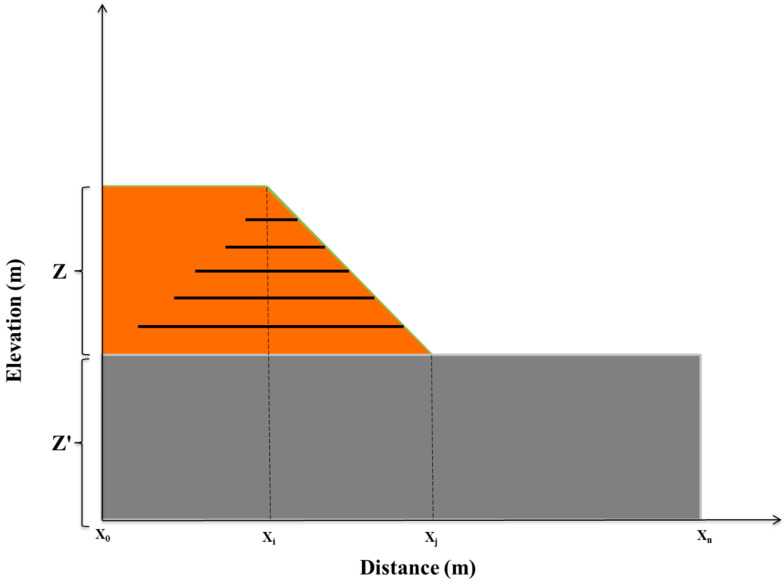
A reinforced slope sample in the study area.

The geometry of the nine GRSs was roughly similar according to the geology of the study area, which was situated on saturated clay. Geotextiles have the same technical properties, since the shear strength properties of the soil in slopes are roughly identical. The slopes, which are located adjacent to highways, were threatened by failure due to their poor drainage systems. As shown in Figure 4, a reinforced slope and its distance from the road were defined as a bound of [X_0_, X_j_] and [X_j_, X_n_], respectively. Z and Z’ represent the heights of the slope and the highway, respectively. For the sake of drainage function or fluid transmission, the geotextile was placed in contact with a material of low permeability, through which water was seeping slowly in all the GRSs. Its function was to gather water and convey it within its plane towards an outlet. The main reason for the failure among all considered GRSs was a poor drainage system. The described characteristics demonstrate the interrelationship among slopes.

### 3.2. GRS Failure Modeling

Probabilistic models incorporate random variables and probability distributions into the model of an event or phenomenon. While a deterministic model gives a single possible outcome for an event, a probabilistic model gives a probability distribution as a solution. Markov chain Monte Carlo (MCMC) simulation comprises a class of algorithms for sampling from a probability distribution. By constructing a Markov chain with the desired distribution as its equilibrium distribution, a sample of the desired distribution can be obtained by recording states from the chain. MCMC can be used to find the posterior distribution and sample it. Thus, it is used to fit a model and draw samples from the joint posterior distribution of the model parameters. Bayesian inference aims to maintain a full posterior probability distribution among a set of random variables. Sampling algorithms based on MCMC techniques can be used to perform inference in such models. To tackle the shortcomings in evaluating the reliability of reinforced slopes, Bayesian inference (BI) and MCMC simulation were employed.

#### Hierarchical Bayesian Model (HBM)

**Figure 5:**
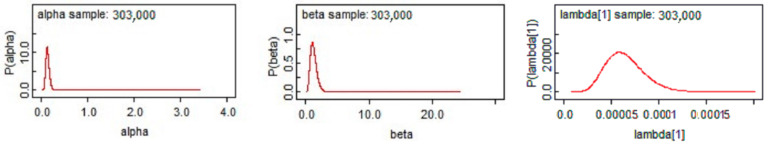
Density functions for α, β, and λ

The failure assessment of the considered GRSs indicates a constant failure rate during their lifetimes. According to the time-independent gathered data and considering the previous study [42], the most efficient approach to estimate the failure rate of nonoverlapping interval events is to employ an HPP. The failure rates of presented GRSs are listed in Table 1. Three chains with the over dispersed initial values of α and β were established to secure the convergence and make an accurate prediction of the modeling procedure. Then, 300,000 iterations were performed for each chain to calculate α and β. The predicted posterior distribution of α, β, and *λ* is shown in Figure 5.

**Figure 6:**
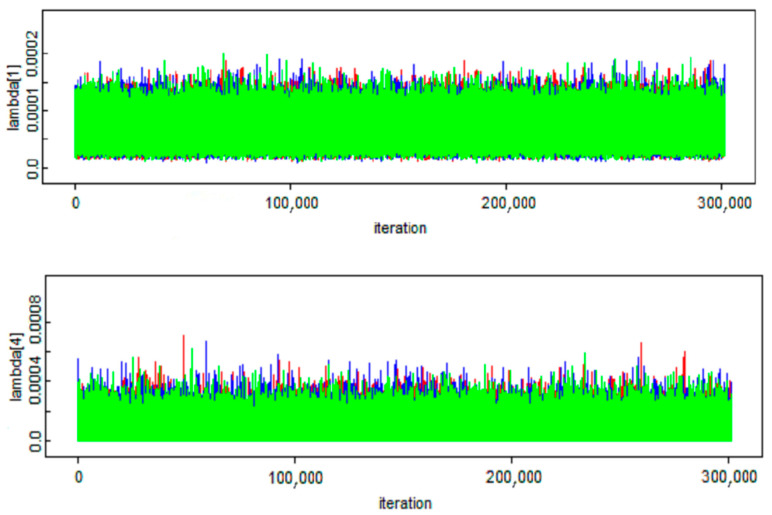
The trace of *λ* in the Markov chain Monte Carlo (MCMC) sampling.

**Figure 7:**
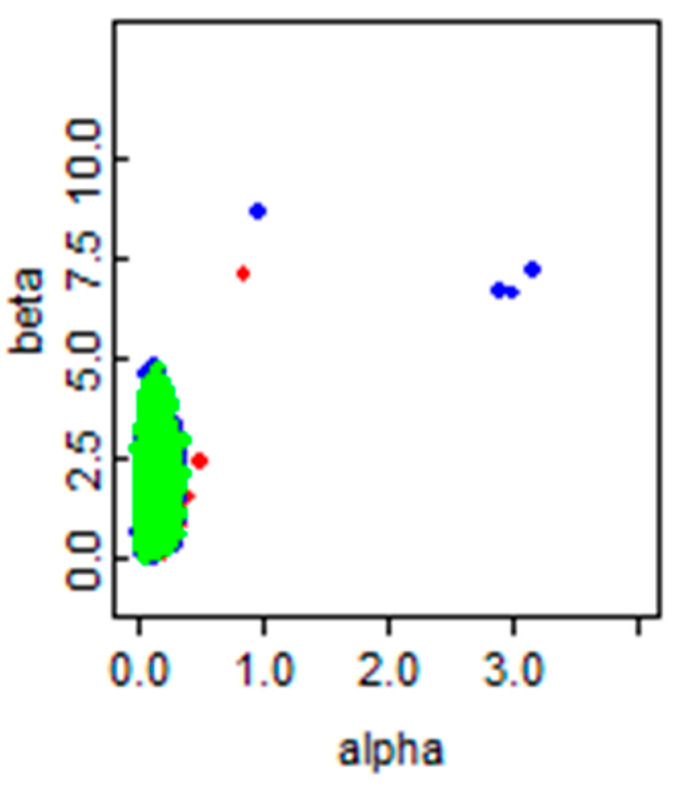
The correlation of α and β.

Figure 6 shows the random iteration history of the parameter of interest in the exponential model (*λ*) in the MCMC modeling over 300,000 iterations. The correlation of the shape and scale parameters (α, β) are plotted in Figure 7. The three distinct colors demonstrate the treatment of the three aforementioned chains in the MCMC simulation [46].

**Figure 8:**
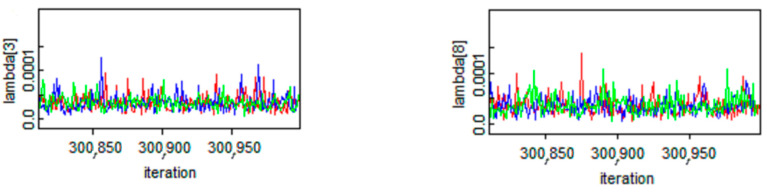
The dynamic trace of *λ* in the MCMC modeling.

In the proposed case study, the posterior mean value of nine *λ* according to the presented reinforced slopes was calculated. Due to space limitations, the dynamic trace of *λ* in two stations is illustrated in Figure 8 to show the convergence of three employed chains in the MCMC simulation.

A hierarchical Bayesian analysis was conducted using the data in Table 1. The posterior predictive distribution (*λ*) was estimated using α and β, which are the shape and the scale parameters for each of the GRSs. The mean posterior value for hyperparameters α  and β were 0.1315 and 1.236, respectively. The maximum failure probability was found for GRS 1 ($6.45\times 10^{-5})$, and the minimum probability was found for GRS 5 ($1.25\times 10^{-6})$. The estimated marginal posterior distributions for α, β, and *λ* for the other GRSs are shown in Table 2.

**Figure 9:**
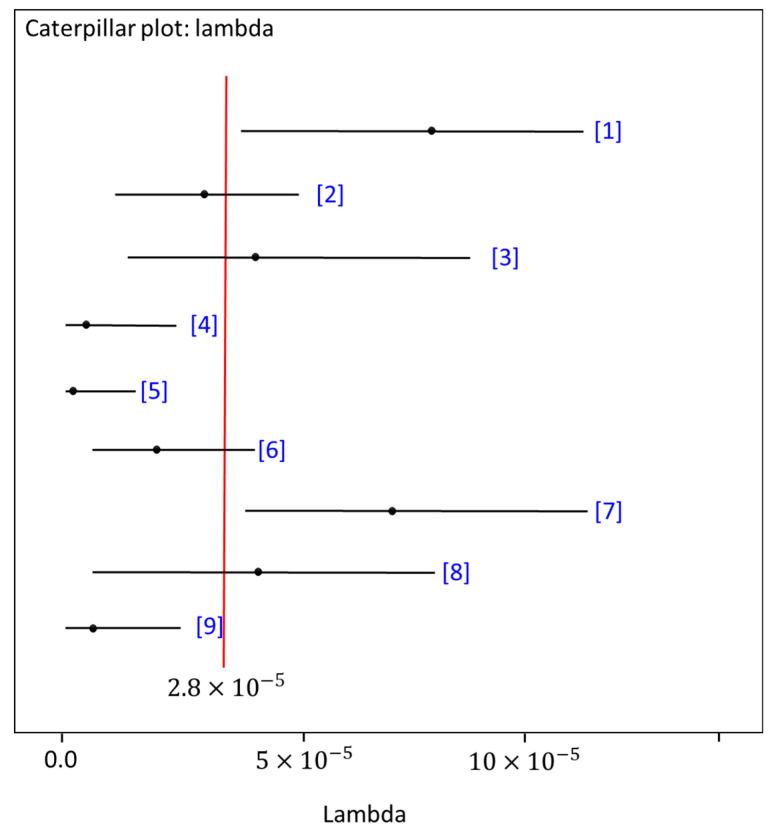
The posterior means and 95% credible intervals for the nine reinforced slopes using hierarchical Bayesian modeling. The black dots denote the posterior mean for each reinforced slope, and the red line is the average of the posterior means.

The 95% credible intervals for *λ* for each source when considering all nine reinforced slopes, based on the Jeffreys prior for *λ* using the obtained data from each source, are depicted in Figure 9 [47]. The mean value of the posterior predictive distribution of *λ* was $2.8\times 10^{-5}$ per hour. Thus, it is likely that the reinforced slopes will experience a failure probability of around $2.8\times 10^{-5}$ during an hour of their lifespans. The Bayesian method can efficiently take advantage of the available data to predict the parameters of the failure model and provide an opportunity to improve asset-management plans. This model can predict the failure, thus directly avoiding unnecessary maintenance and safety consequences.

### 3.3. Prioritizing Maintenance Actions

The developed methodology was capable of estimating the failure probability of the considered GRSs in an integrated system per hour. Note that in evaluating the failure probability of a GRS system, a safe limit condition should be adopted. Maintenance of the considered GRSs should be started when the failure probability exceeds the safe limit. In our study, the prediction for the reinforced slopes shows that a failure probability of approximately $2.8\times 10^{-5}$ is expected in an hour for the whole system during its lifetime. For the predicted failure probability, a safe limit condition of 70,000 h was assumed. Consequently, the maintenance procedure should begin after approximately eight years for the GRS system. The safe limit should be determined for other systems when considering the estimated failure probability.

## 4. Conclusions

This study proposes a methodology for predicting the failure rate of an integrated system of several slopes reinforced with geotextiles. While a wide range of essential components can cause the failure of reinforced slopes, our study selected a poor drainage system as the cause. The installation of a proper drainage system can alleviate the threat of failure. The GRSs had the same geometry, and the technical properties of the geotextiles were the same, since the shear strength of the soil in the slopes was identical. Bayesian inference was employed to model associated risks. The critical advantage of HBM is its flexibility in modeling different groups of phenomena with observations. Additionally, HBM can improve the inference of group data containing lower numbers of observations using the hierarchical model of the group-specific data.

In an HBM, the fluctuation of acquired data is incorporated, and their correlations can lead to predicting the failure mode when considering these uncertainties. To develop the HBM, the total number of failures during the reinforced slopes’ exposure time was assigned. To demonstrate the application of the proposed methodology, a real case study of nine GRSs in different zones of Tehran was performed. Regarding the constant failure rate derived from the performance analysis of the considered GRSs, using a homogenous Poisson process (HPP), a gamma distribution was adopted to estimate the failure rate *λ* that considered the source-to-source variability between the source of data. Prior to calculating *λ*, the hyperparameters including α and β as the first stage prior to distribution were calculated at mean values of 0.1315 and 1.236, respectively. The results show that the whole system is predicted to experience a failure probability of $2.8\times 10^{-5}$ per hour. The proposed time-wise probabilistic model can be adopted to estimate the failure propagation in soil by presenting an unbiased minimum variance category of failure modeling approaches. This method can help city managers assign a safe limit for each GRS based on historical failure data. This modeling is also practical for time and budget constraints and maintenance prioritization in cities with low budgets.

## Figures and Tables

**Table 1:** The slope failure rates due to a poor drainage system during their lifetimes.

Reinforced Slope	No. of Failures	Exposure Time (Hours)
1	10	157,180
2	7	297,240
3	5	139,760
4	1	209,140
5	0	104,819
6	4	262,480
7	11	175,200
8	4	113,860
9	2	254,180

**Table 2:** The statistical properties of the posterior values of the hyperparameters and lambda.

HBM Parameters	Mean	2.5 Percentile	97.5 Percentile
*α*	0.1315	0.07	0.2121
*β*	1.236	0.4576	2.401
*λ*_1_	6.45 × 10^−5^	3.11 × 10^−5^	1.10 × 10^−4^
*λ*_2_	2.40 × 10^−5^	9.76 × 10^−6^	4.47 × 10^−5^
*λ*_3_	3.67 × 10^−5^	1.21 × 10^−5^	7.47 × 10^−5^
*λ*_4_	5.41 × 10^−6^	1.96 × 10^−7^	1.90 × 10^−5^
*λ*_5_	1.25 × 10^−6^	4.05 × 10^−20^	1.12 × 10^−5^
*λ*_6_	1.57 × 10^−5^	4.39 × 10^−6^	3.40 × 10^−5^
*λ*_7_	6.36 × 10^−5^	3.18 × 10^−5^	1.06 × 10^−4^
*λ*_8_	3.63 × 10^−5^	1.02 × 10^−5^	7.86 × 10^−5^
*λ*_9_	8.39 × 10^−6^	1.12 × 10^−6^	2.28 × 10^−5^

## Data Availability

No new data were created or analyzed in this study. Data sharing is not applicable to this article.
